# A comparison of RIFLE with and without urine output criteria for acute kidney injury in critically ill patients

**DOI:** 10.1186/cc11808

**Published:** 2012-10-18

**Authors:** Kama A Wlodzimirow, Ameen Abu-Hanna, Mathilde Slabbekoorn, Robert AFM Chamuleau, Marcus J Schultz, Catherine SC Bouman

**Affiliations:** 1 Department of Medical Informatics, Academic Medical Center, University of Amsterdam, Meibergdreef 9, Amsterdam, 1105 AZ, The Netherlands; 2 Department of Intensive Care, Medisch Centrum Haaglanden, Lijnbaan 32, The Hague, 2512 VA, The Netherlands; 3 Tytgat Institute for Liver and Intestinal Research, Academic Medical Center, University of Amsterdam, Meibergdreef 69-71, Amsterdam, 1105 BK, The Netherlands; 4 Department of Intensive Care, Academic Medical Center, University of Amsterdam, Meibergdreef 9, Amsterdam, 1105 AZ, The Netherlands

## Abstract

**Introduction:**

The Risk, Injury, Failure, Loss, and End-Stage Renal Disease (RIFLE) is a consensus-based classification system for diagnosing acute kidney insufficiency (AKI), based on serum creatinine (SCr) and urine output criteria (RIFLE_SCr+UO)_. The urine output criteria, however, are frequently discarded and many studies in the literature applied only the SCr criteria (RIFLE_SCr_). We diagnosed AKI using both RIFLE methods and compared the effects on time to AKI diagnosis, AKI incidence and AKI severity.

**Methods:**

This was a prospective observational cohort study during four months in adult critically ill patients admitted to the ICU for at least 48 hours. During the first week patients were scored daily for AKI according to RIFLE_SCr+UO _and RIFLE_SCr. _We assessed urine output hourly and fluid balance daily. The baseline SCr was estimated if a recent pre-ICU admission SCr was unknown. Based on the two RIFLE methods for each patient we determined time to AKI diagnosis (AKI-0) and maximum RIFLE grade.

**Results:**

We studied 260 patients. A pre-ICU admission SCr was available in 101 (39%) patients. The two RIFLE methods resulted in statistically significantly different outcomes for incidence of AKI, diagnosis of AKI for individual patients, distribution of AKI-0 and distribution of the maximum RIFLE grade. Discarding the RIFLE urine criteria for AKI diagnosis significantly underestimated the presence and grade of AKI on admission and during the first ICU week (*P *< 0,001) and significantly delayed the diagnosis of AKI (*P *< 0.001). Based on RIFLE_SCr _45 patients had no AKI on admission but subsequently developed AKI. In 24 of these patients (53%) AKI would have been diagnosed at least one day earlier if the RIFLE urine criteria had been applied. Mortality rate in the AKI population was 38% based on RIFLE_SCr _and 24% based on RIFLE_SCr+UO _(*P *= 0.02).

**Conclusions:**

The use of RIFLE without the urine criteria significantly underscores the incidence and grade of AKI, significantly delays the diagnosis of AKI and is associated with higher mortality.

## Introduction

Acute kidney injury (AKI) is a common clinical syndrome in the intensive care unit (ICU) and associated with an increase in morbidity, mortality and length of stay [1]. The Risk, Injury, Failure, Loss and End-Stage Renal Disease (RIFLE) classification system developed in 2004 by the Acute Dialysis Quality Initiative (ADQI) [2,3] is a consensus definition for the diagnosis of AKI. The severity grades risk, injury and failure are defined on the basis of the changes in serum creatinine (SCr) or urine output where the worse of each criterion is used (Table 1). If a reliable baseline SCr is unknown, ADQI suggests the calculation of a theoretical baseline value by the modification of diet in renal disease (MDRD) equation [4]. RIFLE is the first widely accepted AKI definition, validated in over half a million patients worldwide [5-7]; however, the urine criteria are frequently discarded [8-16]. Notably, transient oliguria occur frequently in ICU patients and its use often identifies a higher percentage of AKI patients compared to SCr alone [17-19].

**Table 1:** Risk, Injury, Failure, Loss and End-stage Kidney (RIFLE) classification [2]

Class	Serum creatinine criteria	Urine output criteria
Risk	↑ SCr ≥1.5 × from baseline	<0.5 ml/kg/h ≥6 h
Injury	↑ SCr ≥2 × from baseline	<0.5 ml/kg/h ≥12 h
Failure	↑ SCr ≥3 × from baseline *or *an acute ↑ SCr ≥44 μmol/l from baseline SCr ≥354 μmol/l	<0.3 ml/kg/h ≥24 h *or *anuria ≥12 h

Loss	Complete loss of kidney function >4 weeks	
End-stage	End-stage kidney disease >3 months	

Only one criterion (serum creatinine or urine output) has to be fulfilled to qualify for a specific stage. SCr, serum creatinine

We hypothesized that discarding the urine criteria not only decreases the estimated incidence of AKI but also increases the time to AKI diagnosis.

We determined the time to reach AKI diagnosis (AKI-0) in a heterogeneous ICU population admitted to the ICU for more than 48 hours using both RIFLE methods (with and without urine output). Additionally, we assessed the impact of these two RIFLE methods on the incidence and grading of AKI.

## Materials and methods

### Study design and setting

We performed anonymous analysis of routinely collected clinical data. The Medical Ethics Review Committee of our institution waived the need for informed consent. The study was carried out between April 2009 and August 2009 in the ICU of the Academic Medical Center, a major university hospital in Amsterdam with a 28-bed general, multidisciplinary closed format ICU. During the study period all patients receiving ICU treatment for more than 48 hours were eligible for enrolment. Patients with known end-stage renal disease or receiving renal replacement therapy were excluded.

### Data collection

Demographic data, clinical history (including the lowest documented SCr within six months of ICU admission), and severity of illness were recorded on ICU admission. For each patient the lowest documented SCr within six months of hospital admission was recorded (pre-ICU admission SCr). The estimated SCr baseline was calculated from the MDRD equation assuming a GFR of 75 ml/min/1.73 m^2 ^(MDRD_75_) [4]. Urine output was measured hourly by visual readings of the amount of urine accumulated in a urine metre. Fluid balance, SCr and the presence of renal replacement therapy (RRT) were documented daily. We did not record details of type of fluid administration, use of diuretics and other medications.

### Assessment of acute kidney injury

During the first seven days of ICU treatment patients were scored daily for AKI based on RIFLE using SCr and urine output criteria (RIFLE_SCr+UO_) and based on the RIFLE SCr criteria only (RIFLE_SCr_)_. _The lesser of pre-ICU admission SCr and ICU admission SCr served as baseline renal function. If pre-ICU admission SCr was unknown the baseline was taken as the minimum between the MDRD_75 _based and ICU admission SCr [20].

For each patient we determined the number of days elapsed until AKI was first diagnosed (AKI-0) according to the two RIFLE methods. In addition, we classified patients into four grades according to their maximum RIFLE grade: no AKI, risk, injury and failure. Patients receiving RRT therapy were classified as having failure.

### Statistical analysis

Statistical analyses were performed in the statistical environment R version 2.10.1 (R Foundation for Statistical Computing, Vienna, Austria) [21] and we used the "boot" library for performing the bootstrap procedures. Data are presented as number and percentage, mean ± SD, or median and quartiles. The baseline characteristics of the patients with and without a pre-ICU admission baseline SCr were compared using the t-test (for normally distributed quantities) or the Mann-Whitney U-test and the proportion test (for proportions). We tested differences between the two RIFLE methods for the following outcomes:

a) Difference in the distribution of first day on which AKI was diagnosed

b) Difference in the distribution of the maximum RIFLE grade

To measure the differences in the distributions a) and b), we calculated the 95% confidence interval (CI) around the two-sample Kolmogorov-Smirnov D statistic and the *P*-value associated with the null-hypothesis that D = 0, that is, that there are no differences between the methods for the two distributions. To obtain D's 95% CI we used the standard bootstrap procedure [22] with 3,000 bootstrap samples. A bootstrap sample has the same size as the original dataset and is obtained by random re-sampling, with replacement, from the original dataset. To obtain *P*-values for D we use a permutation test in which we construct 3,000 permutation re-samples and calculate the proportion of times in which the Kolmogorov-Smirnov statistic for the permutation was larger than D.

c) Difference in incidence of AKI and AKI associated mortality

To determine the difference in AKI incidence, that is, having or developing AKI in the first seven days of hospital stay, we again used the basic bootstrap procedure with 3,000 samples [22]. This allowed us to obtain the incidence and variance of AKI in the whole sample in the first seven days of the hospital stay. To test the difference in mortality rate we used the proportion test.

d) Difference in diagnosis of AKI in individual patients

To test differences in concordance between RIFLE methods in diagnosing AKI in individual patients we used the McNemar test. The following example illustrates the difference between incidence of AKI in a sample and AKI diagnosis in individual patients: if method M1 diagnoses three patients as "AKI", "non-AKI" and "AKI", and method M2 diagnoses these same patients as "AKI", "AKI" and "non-AKI" respectively, then the incidence of AKI in both methods is equal (two out of three), but the individual diagnoses are different for the second (non-AKI, AKI) and third (AKI, non-AKI) patient. The diagnoses are hence concordant only in the first patient.

e) Difference in fluid balances

To test differences in fluid balance in patients classified according to both RIFLE methods, we calculated fluid balance both on the first day of AKI diagnosis and cumulative (from ICU admission up to the first day of AKI diagnosis). For comparison we used the Mann-Whitney U-test.

For all analyses, *P *< 0.05 was considered to indicate statistical significance.

## Results

### Patients

During the study period 260 patients were treated in the ICU for at least 48 hours. The demographic data are shown in Table 2. Pre-ICU admission SCr level was available in 101 (39%) and estimated in 159 patients (61%). In the patients with a known prior renal function the difference between pre-ICU admission SCr and estimated baseline SCr was not statistically significant (90 ± 34 μmol/L versus 88 ± 12 μmol/L, *P *= 0.39); however, pre-ICU admission SCr was significantly lower than SCr on ICU admission (90 ± 34 μmol/L versus 125 ± 121 μmol/L, *P *< 0.01). A total of 38 out of 101 patients (38%) had a lower SCr on ICU admission compared to their pre-ICU level (76 ± 40 μmol/L versus 92 ± 38 μmol/L, *P *< 0.0001). In the patients without a known pre-ICU admission SCr the difference between estimated baseline SCr and SCr on ICU admission was statistically significant (88 ± 12 μmol/L versus 107 ± 69 μmol/L, *P *< 0.001). A total of 81 out of 159 (51%) patients had a significantly lower SCr on ICU admission compared to their estimated baseline SCr (64 ± 15 μmol/L versus 90 ± 13 μmol/L, *P *< 0.0001). In the patients with a known pre-ICU admission SCr, the lower of pre-ICU admission and ICU-admission SCr served as the baseline for RIFLE, while in the patients without a known pre-ICU admission, the lower of the estimated SCr and ICU admission SCr served as baseline for RIFLE. Therefore, pre-ICU admission SCr served as baseline for RIFLE in 63 (24%) patients, ICU admission served as baseline for RIFLE in 120 (46%) patients and estimated SCr served as baseline for RIFLE in 77 (30%) patients. Patients with a known pre-ICU admission SCr were statistically significantly older and were more frequently surgical patients than patients without a known pre-ICU admission SCr.

**Table 2:** Baseline characteristics

Variables	All (*n *= 260)	Known pre-ICUadmission SCr (*n *= 101)	Unknown pre-ICU admission SCr (*n *= 159)	*P*-value
Age (years)	60 ± 16	64 ± 13	58 ± 17	<0.01
Men (%)	62	65	60	NS
Weight (kg)	83 ± 22	81 ± 22	84 ± 22	NS
APACHE II	21 ± 8	21 ± 7	21 ± 9	NS
SAPS	52 ± 17	54 ± 16	50 ± 17	NS
Type of admission (%)				
Medical	56	50	61	NS
Surgical urgent	27	22	30	NS
Surgical elective	17	28	9	<0.01
Baseline SCr (μmol/l)				
Pre-ICU admission	-	90 ± 34	unknown	
Admission	115 ± 93	125 ± 121	107 ± 69	NS
Estimated^2) ^	88 ± 12	88 ± 12	88 ± 13	NS
ICU mortality (%)	21	19	22	NS
ICU stay (days)	7.0 (5.0 to 12.0)	7.0 (4.25 to 10.75)	8.0 (5.0 to 13.5)	NS
Comorbidity (%)				
Hypertension	30	34	27	NS
Diabetes mellitus	17	22	14	NS
Chronic Renal Failure^1)^	6	10	4	NS
Cardiovascular disease	33	39	30	NS

Values are mean ± SD, median (quartiles) or percentage of patients. APACHE, Acute Physiology and Chronic Health Evaluation; GFR, glomerular filtration rate; SAPS, Simplified Acute Physiology Score; SCr, serum creatinine. ^1) ^GFR <45 ml/min, based on pre-ICU admission morbid SCr and MDRD equation [4]. ^2) ^Based on the MDRD equation assuming a GFR of 75 ml/min/1.73 m^2 ^[4].

a) Difference in the distribution of first day on which AKI was diagnosed

**Figure 1:**
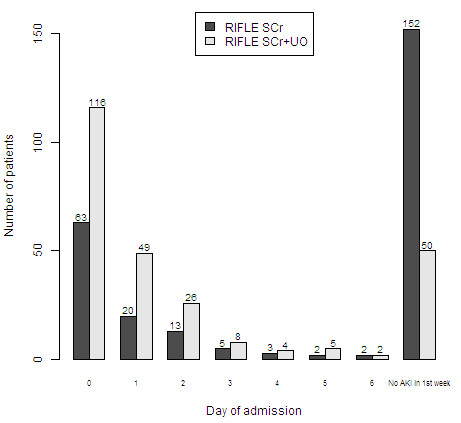
**Distribution of first day on which AKI was diagnosed according to two RIFLE methods**. RIFLE_SCr+UO_, based on serum creatinine and urine criteria; RIFLE_SCr_, based on serum creatinine criteria only.

**Figure 2:**
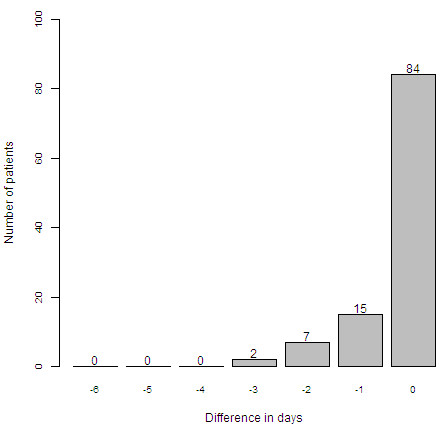
Time benefit of RIFLE_SCR+UO _in patients primarily diagnosed using RIFLE_SCr_

Figure 1 compares the distribution of timing of AKI-0 based on the two RIFLE methods. The difference between the two methods was statistically significant D = 0.39, 95% CI 0.33 to 0.45, *P *< 0.0001. On admission, 116 (45%) patients had AKI based on RIFLE_SCr+UO_, while based on RIFLE_SCr_, only 63 (24%) had AKI. Based on RIFLE_SCr_, 45 patients had no AKI on admission but subsequently developed AKI within seven days of ICU stay. In 24 of these patients (53%), AKI would have been diagnosed at least one day earlier if the RIFLE urine criteria had been applied (Figure 2). During the first ICU week, 102 (39%) patients were diagnosed with AKI based on a reduction in urine output (RIFLE_SC+UO_), but without a rise in SCr, and thus were not diagnosed with RIFLE_SCr_: 38 (15%) patients on admission; 33 patients (13%) on Day 1; 18 (7%) patients on Day 2; 7 (3%) patients on Day 3; 2 (0.8%) patients on Day 4; and 4 (1.5%) patients on Day 5. In 9 (9%) of these patients CVVH was started before a rise in SCr and 8 (8%) patients died without reaching the RIFLE_SCr _criteria. Urine output recovered after one or more days in the remaining 85 (83%) patients.

b) Difference in the distribution of the maximum RIFLE grade

**Figure 3:**
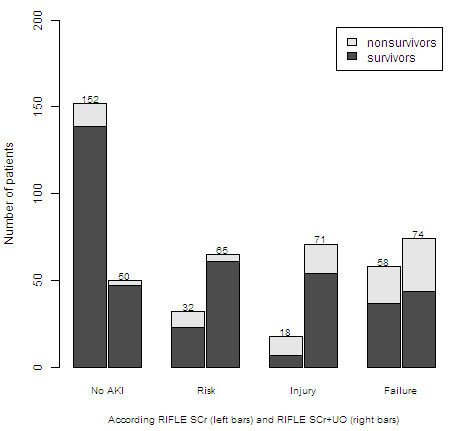
**Distribution of the maximum RIFLE grade and associated mortality based on two RIFLE methods. **RIFLE_SCR+UO_, based on serum creatinine and urine criteria; RIFLE_SCr_, based on serum creatinine criteria only.

Figure 3 compares the two distributions of the maximum RIFLE grade during the first ICU week. The 95% CI around the Kolmogorov-Smirnov D statistic and associated *P*-value D = 0.39, 95% CI 0.33 to 0.45, *P *< 0.0001 show that one method resulted in a significantly different distribution than the other method. RIFLE_SCr _classified 102 (39%) patients more as having no AKI in the first week of ICU-stay comparing to RIFLE_SCr+UO. _Those patients according to RIFLE_SCr+UO _had: AKI-risk 46 (18%) patients, AKI-injury 49 (19%) patients and AKI-failure 7 (3%) patients.

c) Difference in incidence of AKI and AKI-associated mortality

The incidence of AKI in the first ICU week was 42% (95% CI: 36 to 48%), (108 patients) based on RIFLE_SCr _versus 81% (95% CI: 76 to 86%), (210 patients) based on RIFLE_SCr+UO. _95% CI around the difference between two RIFLE methods on AKI incidence (-0.45 to 0.33) shows that the differences were statistically significant, as the CI does not include 0. More non-surviving patients were AKI positive according to RIFLE_SCr+UO _(*N *= 51) than RIFLE_SCr _(*N *= 41); however, the relative mortality rate was significantly higher by RIFLE_SCr _than RIFLE_SCr+UO _(38% versus 24%, *P *= 0.02)

In Figure 3 we presented mortality rates in patients within each RIFLE severity grade.

d) Difference in diagnosis of AKI in individual patients

The difference in diagnosing AKI by the two RIFLE methods is statistically significant (*P *< 0.0001).

e) Difference in fluid balances

The daily fluid balance was calculated using 24-hour fluid intake and output. Based on RIFLE_SCr+UO, _210 patients were diagnosed with AKI of which 174 (83%) patients had a positive fluid balance on AKI-0. Based on RIFLE_SCr_, 108 patients were diagnosed with AKI of which 174 (90%) had a positive fluid balance on AKI-0.

Table 3 shows the 24 hours fluid balance on the first day of AKI diagnosis (AKI-0) as well as the cumulative fluid balance defined as the sum of the daily fluid balances from ICU admission up to and including AKI-0.

**Table 3:** Daily and cumulative fluid balance on first day of AKI diagnosis

AKI-0	AKI based on RIFLE_SCr_		AKI based on RIFLE_SCr+UO_	
	
	Daily^1)^	Cumulative^2)^	Daily^1)^	Cumulative^2)^
Day 0	1,617(620 to 3,348)	1,617(620 to 3,348)	2,217(707 to 3,522)	2,217(707 to 3,522)
Day 1	3,308(1,985 to 5,615)	5,499 *(3,271 to 8,605)	2,581(1,097 to 3,653)	3,587 *(1,287 to 5,588)
Day 2	3,605 *(1,400 to 6,077)	10,547 *(6,565 to 13,796)	981.5 *(81 to 3,196)	4,238 *(1,170 to 7,757)
Day 3	3,353 *(2,106 to 3,532)	13,723 *(13,413 to 17,128)	-528 *(-840 to -96)	4,950 *(2,706 to 5,463)
Day 4	2,137(1,056 to 2,724)	7,965(6,459 to 8,892)	742(372 to 1,563)	5,167.5(1,564 to 8,429)
Day 5	-537.5(-933 to -142)	-495(-509 to -481)	-546(-1,328 to -457)	204(-467 to 3,280)
Day 6	885(548 to 1,222)	-3,732.5(-6,022 to -1,443)	885(548 to 1,222)	-3,732.5(-6,022 to -1,443)

*Statistical significance *P *< 0.05. Values are medians (quartiles); AKI, acute kidney injury; AKI-0, First day of AKI diagnosis, RIFLE_SCr_, RIFLE serum creatinine criteria only; RIFLE_SCr+UO_, RIFLE serum creatinine and urine output criteria. ^1)^Daily fluid balance, fluid balance on first day of AKI-diagnosis; ^2)^Cumulative fluid balance, cumulative fluid balance from ICU admission up to the first day of AKI diagnosis.

f) Continuous veno-venous hemofiltration (CVVH)

Forty-nine patients received CVVH treatment during the first ICU week. The majority of patients (82%) started CVVH within the first three days of ICU admission: 14 patients on ICU admission, 13 patients on Day 1, 13 patients on Day 2, 4 patients on Day 3, 1 patient on Day 4, 2 patients on Day 5 and 2 patients on Day 6. Table 4 shows the maximum RIFLE score before the initiation of CVVH based on the two RIFLE methods. Based on RIFLE_SCr+UO_, all patients had Injury or Failure at the start of CVVH, while based on RIFLE_SCr_, 22 (45%) patients had Injury or Failure, 9 (18%) patients had no AKI and 8 (16%) patients had Risk. The difference between the two RIFLE methods was statistically significant (D = 0.35, 95% CI 0.20 to 0.40, *P *< 0.0001). In seven patients (14%), CVVH was started based on an increased SCr (Injury or Failure) while urine output was not decreased.

**Table 4:** RIFLE scores at the start of continuous veno-venous hemofiltration (number of patients and percentage)

	RIFLE_SCr_	RIFLE_SCr+UO_*
No AKI	9 (18%)	0
Risk	8 (16%)	0
Injury	13 (27%)	16 (33%)
Failure	19 (39%)	33 (67%)
Total	49 (100%)	49 (100%)

*The difference between the two RIFLE methods is statistically significant (D = 0.35, 95% CI 0.20 to 0.40, *P *< 0.0001). RIFLE_SCr_, RIFLE diagnosis based on RIFLE serum creatinine criteria only; RIFLE_SCr+UO_, RIFLE diagnosis based on both serum creatinine and urine criteria; No AKI, patients without any occurrence of RIFLE criteria.

On ICU admission, 14 patients started with CVVH and were, therefore, scored as 'Failure'. Table 5 shows the maximal RIFLE grade on admission based exclusively on SCr and urine output, and not on the presence of CVVH.

**Table 5:** RIFLE scores on the first ICU admission day (number of patients and percentage).

	RIFLE_SCr_	RIFLE_SCr+UO_
No AKI	200 (77%)	144 (55%)
Risk	29 (11%)	54 (21%)
Injury	14 (5%)	36 (14%)
Failure^1)^	17 (7%)	26 (10%)
Total	260 (100%)	260 (100%)
CVVH	14 (5%)	14 (5%)

CVVH, continuous veno-venous hemofiltration; No AKI, patients without any occurrence of RIFLE criteria; RIFLE_SCr_, RIFLE diagnosis based on RIFLE serum creatinine criteria only; RIFLE_SCr+UO_, RIFLE diagnosis based on both serum creatinine and urine criteria. ^1) ^Based on serum creatinine and urine criteria only, the initiation of CVVH not taken into account.

## Discussion

The RIFLE classification is the first widely accepted definition for AKI; however, many studies have applied RIFLE incorrectly without the use of urine output [7]. We performed a prospective observational study and compared AKI diagnosis based on RIFLE_SCr+UO _with that based on RIFLE_SCr. _The two RIFLE methods resulted in statistically significantly different outcomes for incidence of AKI, diagnosis of AKI for individual patients, time to diagnosis of AKI and maximum RIFLE grade. Discarding the RIFLE urine output criteria for AKI diagnosis significantly underestimated the presence of AKI on admission and during the first ICU week (*P *< 0.001), and significantly delayed the diagnosis of AKI (*P *< 0.001). In our study, the use of RIFLE_SCr _instead of RIFLE_SCr+UO _resulted in fewer patients diagnosed with mild AKI (AKI-risk and AKI-injury) and more patients having no AKI. A total of 102 (39%) patients never had AKI during the first ICU week according to RIFLE_SCr_, while these patients were indeed diagnosed as having AKI based on RIFLE_SCr+UO. _The question arises of whether at least some of the oliguric patients without an increase in SCr actually did have AKI, or whether they were oliguric for some other reason (for example, their hydration status) [23,24]. In our patients, AKI-0 was diagnosed based on a decrease in urine output without a rise in SCr in 132 (51%) patients. In 9 (7%) of these patients CVVH was subsequently started before a rise in SCr while in 24 patients (18%) SCr rose in the next one to three days reaching the RIFLE_SCr _criteria. Eight (6%) had persistent oliguria and died without a rise in SCr and 91 (69%) patients recovered and never reached the RIFLE_SCr _Risk criteria. The majority (83%) of patients diagnosed with AKI based on RIFLE_SCr+UO _had positive fluid balances on the day AKI was diagnosed.

These findings suggest that for mild AKI the patient's urine output criterion does not match well with the patient's respective creatinine criterion. Our findings confirm prior observations [19,25]. In the small (*N *= 75) prospective observational study by Macedo *et al*., 28% of patients were diagnosed with AKI based on the SCr criteria only, in comparison to 55% when using only the urine output criteria [25]. In the recent multicentre observational study by Prowle *et al*., AKI diagnosis based on SCr was infrequent, while oliguria was relatively common [19].

In the present study, the applied RIFLE method also affected the time to diagnosis of AKI. In comparison with RIFLE_SCr+UO_, the use of RIFLE_SCr _increased the time to AKI diagnosis and resulted in fewer patients with AKI on admission: 210 (81%) patients had AKI during the first week of ICU according to RIFLE_SCr+UO _while only 108 (42%) patients had AKI according to RIFLE_SCr. _Of note, on the day of ICU admission 63 (24%) patients had AKI according to RIFLE_SCr _while 116 (45%) patients had AKI according to RIFLE_SCr+UO. _According to RIFLE_SCr_, 45 patients developed AKI after ICU admission and in 53% of these patients AKI would have been diagnosed at least one day earlier based on the RIFLE urine criteria. Our findings are congruent with the recent prospective study by Macedo *et al*. in 317 critically ill surgical patients, showing that oliguria diagnosed AKI earlier in comparison with the SCr criterion [26].

Our findings are not surprising. Different definitions lead to different answers. An important factor is why most studies did not apply the recommended consensus urine output criteria [3]. The catalyst for the changes in SCr in the consensus definition came from Chertow's paper: a solid statistical argument [27]. In contrast, the urine output criteria arrived via expert opinion; however, there is always the possibility that it is wrong. In addition, measuring urine output is tedious and it is still unclear how the hourly criteria should be applied (continuously or for each six-hour period of the day), with or without diuretics. Many studies omitted the urine criteria because they retrospectively applied the RIFLE criteria to existing databases that did not capture either any urine output criteria or only captured urine output data in a form that cannot be applied. The big question remains - does it really matter and why? We need to know whether defining AKI with or without including urine output actually leads to a difference in AKI-outcome associations. In the present study, ICU mortality in patients with AKI was significantly higher when AKI was diagnosed by RIFLE_SCr _(38%) compared to that based on RIFLE_SCr+UO _(24%)_. _Similar differences are also suggested by two large multicenter epidemiologic studies by Hoste *et al*. (AKI based on RIFLE_SCr+UO_) and Uchino *et al*. (AKI based on RIFLE_SCr_) [16,20]. In these two studies, baseline mortality in non-AKI patients was comparable; however, mortality in the AKI-risk, -injury and -failure group was much higher in the cohort studied by Uchino *et al*., despite the fact that the latter was a hospital-wide population and the former a general ICU population [16,20]. Similarly, the systematic review by Ricci *et al*. showed that the relative risk for death among studies that used RIFLE_SCr+UO _was lower than in those using RIFLE_SCr _[6]. In the present study, mortality in the Risk and Injury groups was higher when AKI was based on RIFLE_SCr, _while in the Failure group mortality was higher when AKI was based on RIFLE_SCr+UO. _AKI-associated mortality, however, was not part of our primary hypothesis and the small number of patients in each RIFLE stratum keep us from any conclusions.

In addition to its effect on AKI-associated mortality, the nonuse of the urine criterion may also influence the diagnostic accuracy of new biomarkers for AKI, including neutrophil gelatinase-associated lipocalin (NGAL) and cystatin C [11,28-31]. Serum cystatin C was found to be a good predictor for AKI (without urine criteria) in the study by Herget-Rosenthal [11], while cystatin C was a poor predictor for AKI (with urine criteria) in the study by Royakkers *et al. *[31]. In addition to case mix, the opposite findings of both studies may also be caused by the application of two different RIFLE methods (with and without urine output criteria).

To apply the SCr criteria of RIFLE information on prior renal function is needed. When a pre-ICU admission SCr is not available, ADQI suggest that the baseline SCr be estimated from the MDRD formula [2]. Zavada *et al. *showed that estimating baseline SCr may over- or underestimate AKI [32]; however, in another study by Bagshaw *et al. *[33], estimating baseline by the MDRD equation appeared to perform reasonably well for determining the RIFLE categories as long as the pre-ICU admission GFR was near normal. In our study, a pre-ICU admission SCr was available in 101 (39%) patients and in these patients the difference between pre-ICU SCr and estimated SCr was not statistically significant (90 ± 34 μmol/L versus 88 ± 12 μmol/L, *P *= 0.39. However, SCr level on ICU admission was significantly higher than the pre-ICU admission SCr (125 ± 121 μmol/L versus 90 ± 34 μmol/L, *P *< 0.01). Of note, in the present study, 81 (51%) out of the 159 patients with an unknown prior SCr had lower SCr at ICU admission compared with the estimated SCr. Although this issue is not discussed by the ADQI, in these patients we used the lower SCr level as suggested by Hoste *et al. *[20].

In the present study patients receiving CVVH were classified as Failure as suggested by the acute kidney injury network (AKIN) [3]; however, in the original RIFLE system introduced by the ADQI, renal replacement therapy was not included as a distinct stage [2]. Indeed, it may be questionable to classify patients as Failure if they did not achieve the specific RIFLE score. In our study, using RIFLE_SCr+UO, _67% of the patients had Failure and 33% had Injury at the start of CVVH. In contrast, using RIFLE_SCr, _only 39% of the patients had Failure, 27% had Injury, 16% had Risk and 18% had no AKI. Given the variability in the timing of renal replacement therapy worldwide, it may be more appropriate to always report the AKI stage at the start of renal replacement therapy in future epidemiologic studies.

Our study is the first study comparing the effects of two RIFLE methods (with and without urine output criteria) on time to AKI diagnosis as well as AKI incidence, AKI associated mortality and maximum AKI grade. We, however, recognize the limitations of our study. First, our study is single-centre, including a limited number of patients. Second, SCr was measured daily, while urine output was measured hourly. More frequent SCr measurements may result in earlier detection of AKI. Third, although we recorded fluid status, we did not evaluate whether our patients received diuretics. However, although the use of diuretics is common practice worldwide, their use is not explicitly addressed in the RIFLE criteria. Fourth, we did not correct SCr for hemodilution. A positive fluid balance may cause dilution of SCr and, therefore, a delay in the diagnosis based on RIFLE_SCr _[18]. Two studies showed that hemodilution of SCr may affect AKI diagnosis [18,34]. The basis for the development of the RIFLE classification, however, was Chertow's paper [27] showing that a small rise in SCr increased mortality, and this paper did not correct for hemodilution. In addition, estimating the dilution factor in critically ill patients is notoriously difficult. Fifth, we did not specifically evaluate patients with chronic kidney disease because this subgroup was too small in our sample. Last, our results were statistically significant; however, future research will need to study the clinical significance in more detail.

## Conclusions

Although the RIFLE classification is meant to provide a uniform AKI definition, at least two RIFLE methods (with and without urine output criteria) are used in the literature. In the present study, comparison of the two methods showed statistically significant differences in time to diagnosis of AKI, AKI incidence, AKI associated mortality and maximum AKI grade. Discarding the urine output criteria delayed the diagnosis of AKI, decreased the incidence of AKI diagnosis and was associated with higher mortality.

Our findings suggest that, even when the 'consensus' RIFLE definition is used, the methods employed for estimating AKI need to be robustly reported, and that most already published AKI retrospective epidemiological studies may, therefore, be inaccurate.

## Key messages

Use of RIFLE without the urine criteria significantly:

• underscores the incidence of AKI,

• underscores severity of AKI,

• delays the diagnosis of AKI,

• is associated with higher mortality

## Abbreviations

ADQI Acute Dialysis Quality Initiative; AKI: acute kidney insufficiency; AKI-0: first day of AKI diagnosis; AKIN: acute kidney injury network; APACHE: Acute Physiology and Chronic Health Evaluation; CVVH: continuous veno-venous hemofiltration; GFR: glomerular filtration rate; ICU: intensive care unit; MDRD: modification of diet in renal disease; NGAL: neutrophil gelatinase-associated lipocalin; RIFLE: risk, injury, failure, loss, and end-stage renal disease classification; RIFLE_SCr_: RIFLE based on serum creatinine criteria only; RIFLE_SCr+UO_: RIFLE based on serum creatinine and urine output criteria; RRT: renal replacement therapy; SAPS: Simplified Acute Physiology Score; SCr: serum creatinine

## Competing interests

The authors declare that they have no competing interests.

## Authors' contributions

KW helped in acquisition of data, performed the statistical analysis, interpreted the results and drafted the manuscript. AAH supervised and performed parts of the statistical analysis, interpreted the results and was involved in critically revising the manuscript. MS participated in acquisition of data and revision of the manuscript. RC and MJS were involved in critically revising the manuscript. CB conceived the study, participated in the design of the study and acquisition of data, interpreted the results and drafted the manuscript. All authors read and gave final approval of the version of the manuscript to be published.
